# DEAD-box helicase 27 promotes colorectal cancer growth and metastasis and predicts poor survival in CRC patients

**DOI:** 10.1038/s41388-018-0196-1

**Published:** 2018-03-14

**Authors:** Jieting Tang, Huarong Chen, Chi-Chun Wong, Dabin Liu, Tong Li, Xiaohong Wang, Jiafu Ji, Joseph JY Sung, Jing-Yuan Fang, Jun Yu

**Affiliations:** 1 Institute of Digestive Disease and Department of Medicine and Therapeutics, State Key laboratory of Digestive Disease, Li Ka Shing Institute of Health Sciences, CUHK Shenzhen Research Institute, Prince of Wales Hospital, The Chinese University of Hong Kong, Hong Kong, China; 2 State Key Laboratory for Oncogenes and Related Genes, Key Laboratory of Gastroenterology & Hepatology, Ministry of Health, Division of Gastroenterology and Hepatology, Ren Ji Hospital, School of Medicine, Shanghai Jiao Tong University, Shanghai Cancer Institute,Shanghai Institute of Digestive Disease, 145 Middle Shandong Road, Shanghai, 200001 China; 3 Department of Surgery, Peking University Cancer Hospital and Institute, Beijing, 100142 China

## Abstract

Copy number alterations (CNAs) are crucial for colorectal cancer (CRC) development. In this study, DEAD box polypeptide 27 (DDX27) was identified to be highly amplified in both TCGA CRC (474/615) and primary CRC (47/103), which was positively correlated with its mRNA overexpression. High DDX27 mRNA (*N* = 199) and protein expression (*N* = 260) predicted poor survival in CRC patients. Ectopic expression of DDX27 increased CRC cells proliferation, migration and invasion, but suppressed apoptosis. Conversely, silencing of DDX27 exerted opposite effects in vitro and significantly inhibited murine xenograft tumor growth and lung metastasis in vivo. Up-regulation of DDX27 enhanced and prolonged TNF-α-mediated NF-κB signaling. Nucleophosmin (NPM1) was identified as a binding partner of DDX27. DDX27 increased nuclear NPM1 and NF-κB-p65 interaction to enhance DNA binding activity of NF-κB. Silencing NPM1 abrogated DDX27-activating NF-κB signaling and its tumor-promoting function. Together, DDX27 is overexpressed and plays a pivotal oncogenic role in CRC.

## Introduction

Colorectal cancer (CRC) is a major cause of cancer morbidity and mortality worldwide, accounting for 1.4 million new cases and 0.7 million deaths in 2012 [1]. Emerging evidence indicates that CRC is a heterogeneous disease, arising through accumulation of genetic and epigenetic alterations with three major molecular pathways involved: chromosomal instability (CIN) (50–60%), microsatellite instability and the CpG island methylator phenotype [2]. CIN is a hallmark of CRC, resulting in copy number alterations (CNAs) [3]. CNAs are somatic changes of chromosome structure that lead to gain or loss in copies of DNA sections ranging in size from kilobases to megabases [4]. The stepwise accumulation of CNAs confers growth advantage and metastatic competence on tumor cells, thus playing a crucial role in CRC initiation and progression [5]. DNA copy number gain frequently activates oncogenes [6, 7], whereas tumor suppressors are often found in deleted genomic segments [8, 9]. Recent studies have suggested that genes with CNAs are potential biomarkers and/or therapeutic targets for CRC [5]. Thus, it is of great importance to identify gains or losses of specific genes that contribute to CRC development.

By analyzing CRC genomic data from The Cancer Genome Atlas (TCGA) [10], we demonstrated for the first time the frequent gain of copy number of DEAD-box helicase 27 (DDX27) in CRC. DDX27 belongs to the DEAD box nucleic acid helicase family, which is characterized by a conserved “DEAD-box” sequence motif [11]. DEAD box proteins are highly multifunctional with causative role in carcinogenesis [12]. DDX1 is a positive regulator of cyclin-D2 and indispensable for testicular tumorigenesis [13]. DDX3 promotes breast tumorigenesis through inducing epithelial-mesenchymal-like transition, increasing cell motility and invasion [14]. DDX5 and DDX17 contribute to CRC progression by activating β-catenin signaling in colon cancer [15]. Nevertheless, the function of DDX27 remains largely uncharacterized, especially in CRC. In this study, we investigated its functional significance, molecular mechanism and clinical implication.

## Results

### DDX27 is frequently amplified in CRC

**Fig. 1:**
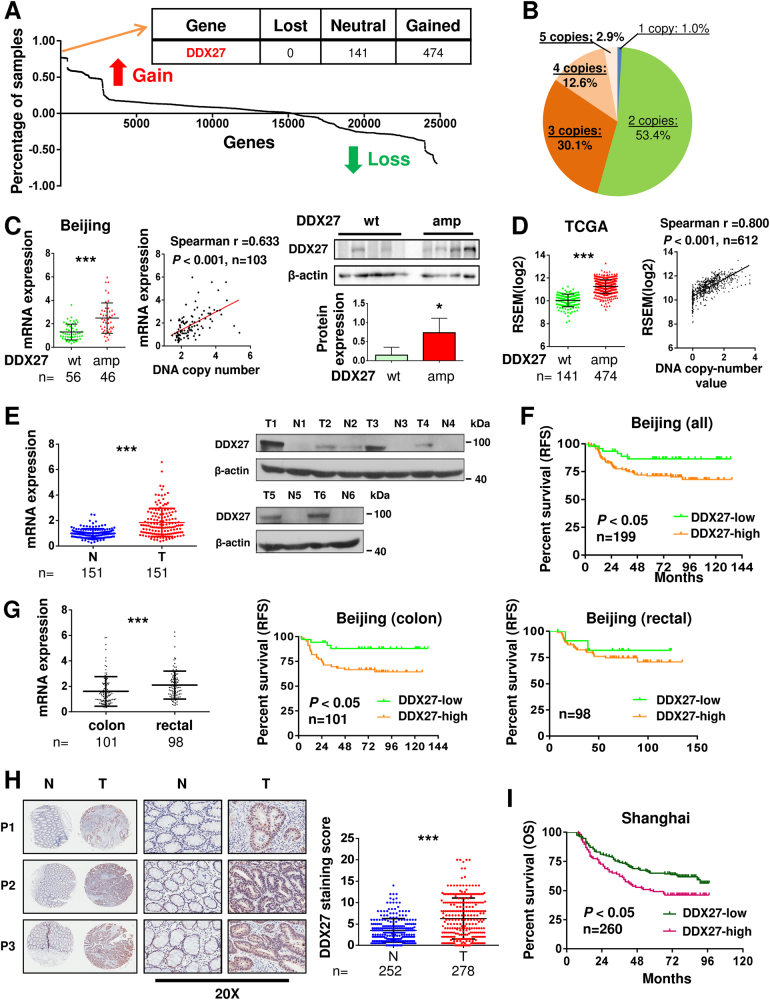
Copy number gain of DDX27 contributed to mRNA overexpression in human CRC. **a** Data of CNA across 24776 genes of 615 TCGA CRC samples. Gene-level frequency of copy number gain and loss was calculated and ranked. Percentage of samples (y axis label of the graph) of individual gene was determined by subtracting frequency of copy number loss from frequency of copy number gain. List of top ranked genes (*n* = 23) with the highest frequency of copy number gain was shown in Table S1. **b** CNAs of DDX27 in 103 primary CRC tissues from Beijing cohort. **c** Comparison of DDX27 mRNA expression (left panel) and protein expression (right panel) between CRC without DDX27 CNA (wt) and CRC with DDX27-amplified (amp) in Beijing cohort. Middle panel showed the correlation between DNA copy number and mRNA expression of DDX27. **d** Left panel showed comparison of DDX27 mRNA expression between CRC without DDX27 CNA (wt) and CRC with DDX27-amplified (amp) in TCGA cohort. Right panel showed the correlation between DNA copy number and mRNA expression of DDX27 in CRC. **e** The mRNA expression of DDX27 in 151 pairs of CRC (T) and adjacent normal tissue (N) (left panel), and protein expression of DDX27 in 6 pairs of primary CRC (T) and adjacent normal tissue (N) (right panel) from Beijing cohort were detected. **f** Kaplan–Meier survival analysis indicated that CRC patients with high expression of DDX27 had worse relapse-free survival in Beijing cohort. **g** Left panel showed differential expression of DDX27 mRNA between colon cancer and rectal cancer in Beijing cohort. Kaplan-Meier survival analysis of DDX27 in predicting relapse-free survival for patients with colon cancer (middle panel) or rectal cancer (right panel) was exhibited. **h** Representative images of DDX27 protein expression in CRC tumor tissues (T) and adjacent normal tissue (N) of three patients (P1-P3) from Shanghai cohorts by immunohistochemistry. Staining score of DDX27 was quantified accordingly. **i** Stratification of the patients in Shanghai cohort according to DDX27 staining score showed that high DDX27 expression (score ≥ 8) predicted poor overall survival (right panel). (*P < 0.05; **P < 0.01; ***P < 0.001)

To identify novel genes with CNAs that are associated with CRC, we comprehensively examined gene-level frequency of copy number gain and loss in 615 human CRC samples from TCGA [10]. Among 24776 genes, DDX27 was identified as one of top ranked genes with the highest frequency of copy number gain (77.1%, 474/615; Fig. 1a and Table S1). On the other hand, analyses of exome sequencing data from TCGA-CRC samples revealed that DDX27 was only mutated in 2.7% of CRC (6/223), of which two were frameshift deletions and the other four were missense mutations (Table S2). To verify the amplification of DDX27 in CRC, we evaluated DDX27 copy number in an independent cohort using TaqMan copy number assay. Consistent with TCGA data, high frequency of DDX27 copy number gain (>2 copies) was observed (45.6%, 47/103; Fig. 1b).

### DNA copy number gain contributes to DDX27 overexpression in CRC

We examined the correlation between DDN27 copy number and mRNA expression. In both Beijing cohort and TCGA cohort, CRC tissues harboring DDX27 amplification exhibited significantly higher mRNA expression as compared to those harboring wild-type DDX27 (without CNA) (both *P < *0.001; Figs. 1c, d). In Beijing cohort, higher DDX27 protein expression in CRC tissues harboring DDX27 amplification was also observed (*P < *0.05; Fig. 1c). Furthermore, DDX27 mRNA expression was positively correlated with DNA copy number value in CRC samples of these two cohorts (both *P < *0.001; Figs. 1c, d). Therefore, gain of copy number is likely a major mechanism that contributes to the up-regulation of DDX27 in CRC.

We next assessed DDX27 expression among various human cancers. Analyses of TCGA gene expression profiling showed significant up-regulation of DDX27 in tumors as compared to adjacent normal tissues in most of human cancer types, especially in CRC (Fig. S1A **and** Table S3). Overexpression of DDX27 at mRNA and protein level was validated in 151 and 6 pairs of primary CRC as compared to adjacent normal tissues, respectively (*P < *0.001; Fig. 1e). In keeping with this, immunohistochemical analysis of 278 CRC and 252 adjacent normal tissues confirmed its overexpression at protein level (*P < *0.001; Fig. 1h).

### High DDX27 expression predicts adverse prognosis in CRC patients

Clinical implication of DDX27 in CRC was investigated. In the Beijing cohort, Kaplan–Meier curves revealed that higher DDX27 mRNA expression (optimal cutoff value was determined by the Cutoff Finder tool) was significantly associated with worse relapse-free survival in patients with CRC (*N* = 199, *P < *0.05; Fig. 1f). By multivariate Cox regression analysis, high DDX27 expression was found to be an independent prognostic factor that predicted shorter relapse-free survival (*P < *0.05; hazard ratio: 2.667; 95% CI, 1.116–6.374; Table S6). In Beijing cohort, DDX27 expression was independent of age, sex and TNM classification, but was found to be associated with tumor site (colon or rectum) (Table S4). Lower DDX27 expression was found in colon cancer than in rectal cancer (*P < *0.001; Fig. 1g), an observation verified in TCGA cohort (*P < *0.001; Fig. S1B). Nevertheless, expression of DDX27 in both colon and rectal cancer was significantly higher than in adjacent normal tissues (both *P < *0.001; Fig. S1A). We stratified the cohort according to tumor site and DDX27 expression. This analysis showed that within colon cancer, patients whose tumor exhibited high DDX27 expression had a shorter survival (*P < *0.05; Fig. 1g). In contrast, there was no statistically significant difference for the survival of patients with rectal cancer between DDX27-high and -low groups (Fig. 1g). Consistent result was obtained in TCGA cohort, in which higher expression of DDX27 predicted poor relapse-free survival in patients with colon cancer (*P < *0.01; Fig. S1C). DDX27 expression in primary colon cancer was an independent prognostic marker for survival in both Beijing cohort (*P < *0.05; hazard ratio: 3.362; 95% CI, 1.157 to 9.771; Table S7 and S8) and TCGA cohort (*P < *0.05; hazard ratio: 7.345; 95% CI, 1.014 to 53.203; Table S9 and S10**)**. Prognostic value of DDX27 in primary colon cancer was further validated in a third cohort. Our result demonstrated that higher staining density of DDX27 (score ≥ 8) (Fig. S1D) in primary colon cancer tissues predicted poor overall survival for patients in Shanghai cohort (*N* = 260, *P < *0.05; Fig. 1i), although this association is not significant by multivariate analysis (*P = *0.080; hazard ratio: 1.389; 95% CI, 0.962–2.005; Table S11). Moreover, high DDX27 staining predicted poor overall survival for patients with early stage (I and II) colon cancer in Shanghai cohort, as evidenced by Kaplan-Meier survival analysis (*P < *0.05, Fig. S1E) and multivariate analysis (*P < *0.05; hazard ratio: 1.860; 95% CI, 1.064–3.250; Table S12). Taken together, DDX27 overexpression is an adverse prognostic factor for CRC patients. DDX27 is located on chromosome 20q, where frequent DNA copy number occurs and correlates with colorectal carcinogenesis. We also evaluated the association between DDX27 and molecular characteristics of colorectal cancer, as defined by TCGA (CIN, invasive, and microsatellite instability/CpG island methylator phenotype (MSI/CIMP) [10]. DDX27 was indeed overexpressed in CIN as compared to invasive and MSI/CIMP subtype (Fig. S1F).

### DDX27 promotes CRC cell growth and inhibits apoptosis

**Fig. 2:**
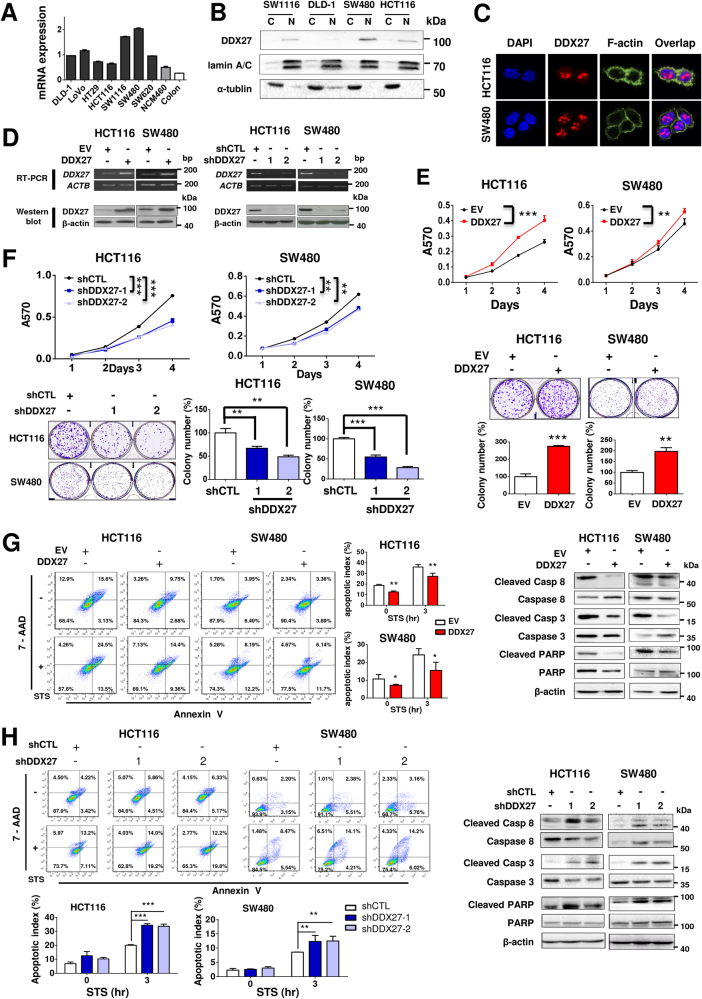
DDX27 promoted CRC cells proliferation and inhibited apoptosis. **a** The mRNA expression of DDX27 in seven CRC cell lines (DLD-1, LoVo, HT29, HCT-116, SW1116, SW480, SW620), a normal human colon epithelial cell line (NCM460) and human normal colon tissue was shown. **b** Western blot analysis of isolated nucleus and cytoplasm from four different CRC cell lines indicated that DDX27 was mainly localized in nucleus. Lamin A/C and α-tublin were used as nuclear marker and cytoplasmic marker, respectively. **c** Immunofluorescence staining for DDX27 (red), F-Actin (green) and DAPI (blue) was shown using HCT116 and SW480 cells. **d** HCT116 and SW480 were stably transduced with lentiviral vectors carrying DDX27 cDNA or DDX27-specific small hairpin RNAs (shDDX27). Accordingly, control groups were transduced with lentiviral carrying empty vector (EV) or negative control shRNA (shCTL). Overexpression or silencing of DDX27 was confirmed by RT-PCR (Top panel) and western blot analysis (Bottom panel). **e** Ectopic expression of DDX27 significantly promoted cell viability by MTT assay (top panel) and increased colony numbers (bottom panel) as compared to control groups in HCT116 and SW480. **f** Knockdown of DDX27 significantly reduced cell viability by MTT assay (top panel) and decreased colony numbers (bottom panel) as compared to controls in HCT116 and SW480. **g** and ** h** HCT116 and SW480 cells with altered expression of DDX27 (overexpression or silencing) were treated with STS (1 and 1.5 uM, respectively). Apoptosis was analyzed by flow cytometry. The apoptotic index in the right panel was defined as the percentage of apoptotic cells. After STS treatment for 3 h, expression of the cleaved form and total level of caspase-8, caspase-3 and PARP was detected by western blot analysis. (*P < 0.05; **P < 0.01; ***P < 0.001)

We determined the mRNA expression of DDX27 in various colon cancer cell lines by qPCR. DDX27 was readily expressed in all investigated CRC cell lines (DLD-1, LoVo, HT29, HCT-116, SW1116, SW480, SW620), but was weakly expressed in normal human colon epithelial cell line (NCM460) and human normal colon tissue (Fig. 2a). DDX27 mainly localized in the nucleus of CRC cell lines, as indicated by western blot analysis (Fig. 2b). The nuclear localization of DDX27 was further confirmed by immunofluorescence staining (Fig. 2c). To characterize the biological role of DDX27, we overexpressed or silenced DDX27 in HCT116 and SW480 cells with lentiviral vectors carrying DDX27 complementary DNA (cDNA) or DDX27-specific small hairpin RNAs (shDDX27), respectively. Controls were transduced with lentiviral carrying empty vector (EV) or negative control shRNA (shCTL). Ectopic expression or silencing of DDX27 was confirmed by reverse transcription polymerase chain reaction and western blot analysis (Fig. 2d). The up-regulation of DDX27 significantly increased cell viability (*P < *0.01) and clonogenicity (*P < *0.01) in both HCT116 and SW480 cell lines as compared to controls (Fig. 2e). In contrast, silencing of DDX27 reduced cell proliferation (*P < *0.01) and colony formation (*P* < 0.01) (Fig. 2f). Next, we assessed the effect of DDX27 on cell apoptosis. Our results demonstrated that ectopic expression of DDX27 led to a significant reduction in apoptosis with decreased expression of the active (cleaved) forms of caspase-8, caspase-3 and poly (ADP-ribose) polymerase (PARP) in both HCT116 and SW480 cells, with and without the presence of staurosporine, an apoptosis inducing drug (Fig. 2g). Conversely, DDX27 depletion sensitized CRC cells to staurosporine treatment as evidenced by increased number of apoptotic cells (*P* < 0.01) and elevated expression of apoptotic markers (Fig. 2h).

**Fig. 3:**
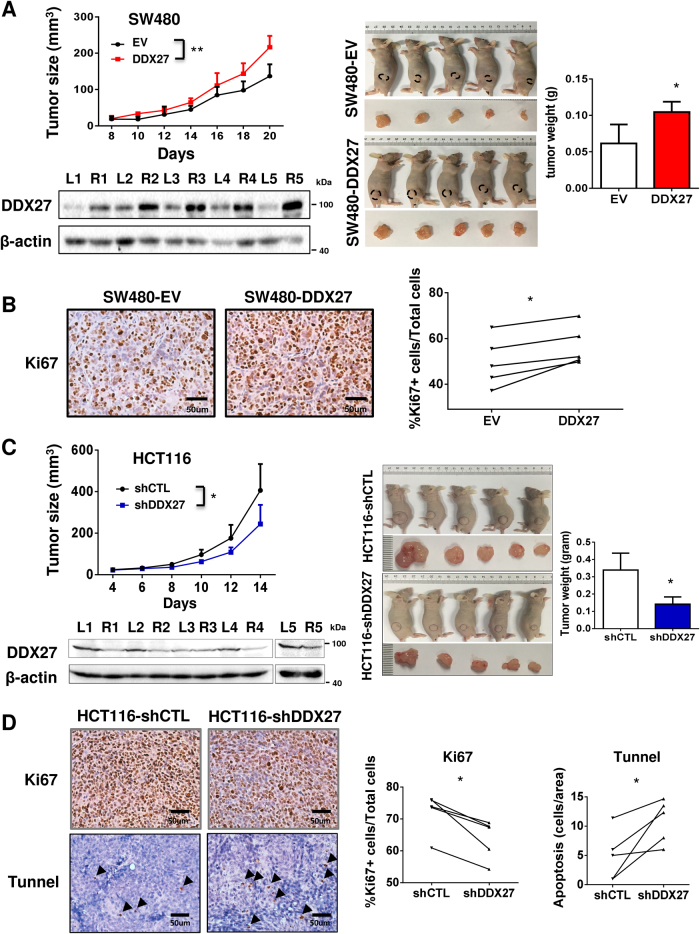
DDX27 exerted oncogenic function in subcutaneous CRC xenograft mouse models. **a** Ectopic expression of DDX27 accelerated growth of SW480 xenografts in nude mices (*N* = 5) as compared to controls (left panel). Right panel showed image of mices (*N* = 5) with tumors and histogram of xenograft tumor weight. Ectopic expression of DDX27 in tumors from SW480-DDX27 groups was confirmed by Western blot (bottom panel). **b** Representative images of Ki67-positive proliferating cells. **c** Knockdown of DDX27 decelerated growth of HCT116 xenografts in nude mices (*N* = 5) as compared to controls (left panel). Right panel showed representative images of HCT116 xenograft tumors and histogram of tumor weight. Knockdown of DDX27 in tumors from HCT116-shDDX27 groups was confirmed by Western blot (bottom panel). **d** Representative images of Ki67-positive (Top panel) and TUNEL-positive cells (Bottom panel). At least 5 fields per slide and 3 slides per animal were counted at 200× magnification. (*P < 0.05; **P < 0.01; ***P < 0.001)

To further investigate the tumourigenic ability of DDX27, SW480-EV and SW480-DDX27 cells were subcutaneously injected into the left and right dorsal flanks of nude mice, respectively. As shown in Fig. 3a, ectopic expression of DDX27 significantly accelerated tumor growth in nude mice (*P < *0.01). The mean tumor weight of the SW480-DDX27 group was increased as compared with SW480-EV group (*P* < 0.05). Overexpression of DDX27 in tumors was confirmed by western blot. In concordance with these findings, more proliferating cells were detected in SW480-DDX27 xenografts, as indicated by Ki-67 assay (*P* < 0.05; Fig. 3b). On the contrary, DDX27 knockdown markedly reduced size and weight of HCT116 xenografts in nude mice (Fig. 3c). Significantly suppressed cell proliferation concomitant with increased apoptosis were observed in HCT116-shDDX27 xenografts, as demonstrated by Ki-67 and TUNEL assays, respectively (both *P* < 0.05; Fig. 3d). Collectively, these data imply that DDX27 plays a pivotal oncogenic role in colorectal carcinogenesis through promoting cell proliferation and inhibiting apoptosis.

### DDX27 increases metastatic ability of CRC cells

**Fig. 4:**
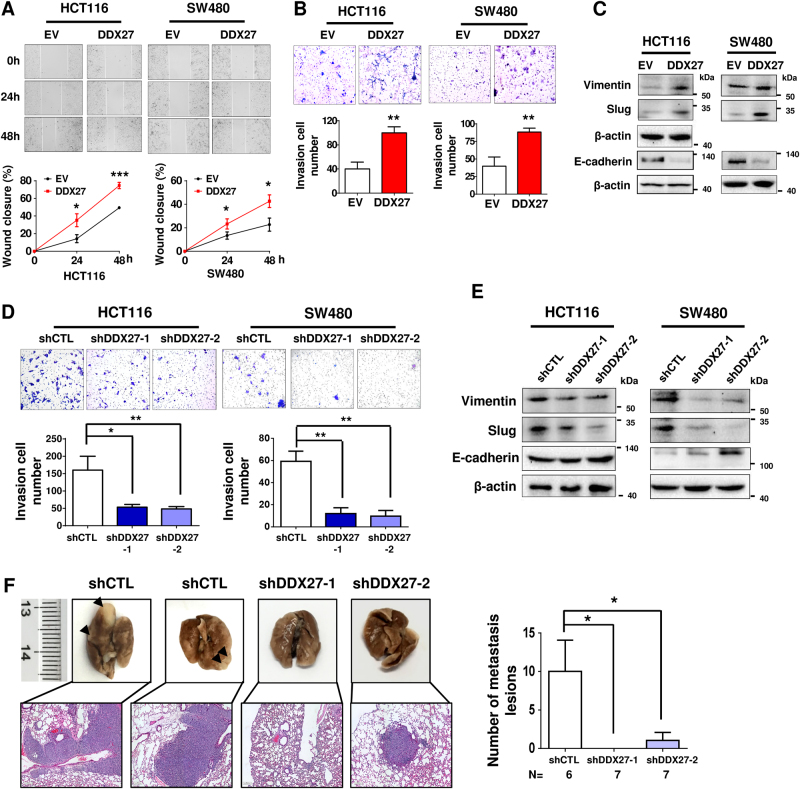
DDX27 promoted metastatic ability of CRC cells. **a** Representative images of wound-healing assay indicated that ectopic expression of DDX27 promoted cell migration in HCT116 and SW480. **b** Representative images of matrigel invasion assay revealed that ectopic expression of DDX27 promoted CRC cells invasion. **c** Western blot analysis revealed that ectopic expression of DDX27 increased expression of mesenchymal markers (Slug and Vimentin) while decreased expression of epithelial markers (E-cadherin). **d** Representative images of matrigel invasion assay revealed that silencing of DDX27 reduced CRC cells invasion. **e** DDX27 silencing decreased expression of mesenchymal markers (Slug and Vimentin) while increased expression of epithelial markers (E-cadherin) as indicated by Western blot analysis. **f** Representative macroscopic appearances of lung metastasis are shown (tumor nodules were indicated as black arrows in top panel). Representative hematoxylin-eosin stained images of the lungs were shown in the bottom panel. Quantification of lung metastatic lesions in HCT116-shCTL (*N* = 6), HCT116-shDDX27-1 (*N* = 7) and HCT116-shDDX27-2 (*N* = 7) groups. Data was expressed as mean ± S.E.M. Student’s *t*-test was performed. (*P < 0.05; **P < 0.01; ***P < 0.001)

We investigated the effect of DDX27 on metastatic ability of CRC cells using in vitro migration and invasion assays, as well as in vivo murine lung metastases model. Ectopic expression of DDX27 significantly promoted cell migration (*P < *0.001) and invasive capability (*P < *0.01) in HCT116 cells compared to control cells (Fig. 4b). Similar results were obtained in SW480 cells (Figs. 4a, b). Western blot analyses showed that DDX27 regulated the epithelial-mesenchymal transition (EMT) through up-regulation of mesenchymal markers (Slug and Vimentin) and down-regulation of epithelial markers (E-cadherin) (Fig. 4c). In contrast, silencing of DDX27 in HCT116 and SW480 cells significantly reduced cell invasion (Fig. 4d), suppressed mesenchymal markers (Slug and Vimentin) and increased epithelial markers (E-cadherin) (Fig. 4e). Furthermore, in vivo murine lung metastases model was established by injecting HCT116 cells via the lateral tail vein. Silencing of DDX27 in HCT116 cells significantly reduced the number of metastatic lesions in the lungs (*P < *0.05; Fig. 4f). These data suggest that DDX27 promotes metastatic ability of colorectal tumor cells.

### DDX27 enhances and prolongs NF-кB signaling

**Fig. 5:**
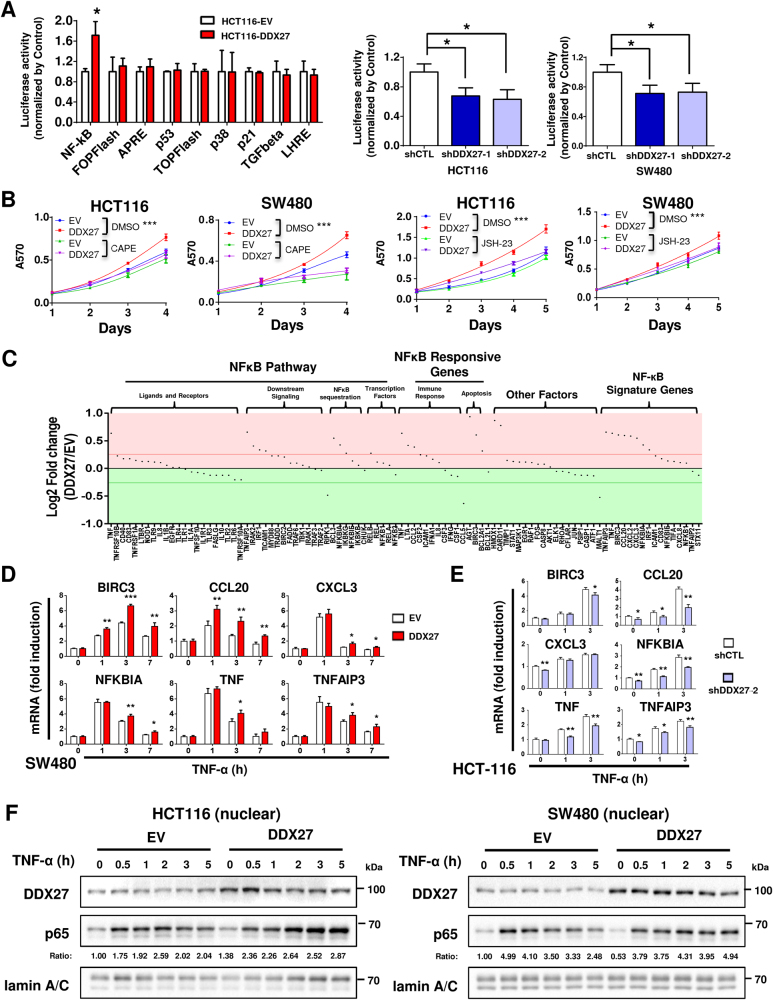
DDX27 enhanced and prolonged NF-кB signaling. **a** In left panel, different cancer pathway reporters consisting of specific pathway-focused transcription factor-responsive firefly luciferase construct were separately transfected to HCT116 cells along with renilla luciferase reporter as internal control. Right panel showed knockdown of DDX27 significantly inhibited NF-кB luciferase reporter activity in both HCT116 and SW480 cells. Reporter activity was determined as the ratio of firefly to Renilla luciferase activity. **b** Caffeic acid phenethyl ester (CAPE) or 4-methyl-1-N- (3-phenylpropyl) benzene-1,2-diamine (JSH-23) was added to cells at indicate concentration (For HCT116, 2.5 mg/L CAPE or 10 uM JSH-23 was used; For SW480, 10 mg/L CAPE or 20 uM JSH-23 was used). Control groups were treated with an equivalent dilution of DMSO. Cell viability was assessed by MTT assays. In both experimental groups (CAPE or JSH-23) and control groups (DMSO), the difference between growth rates of cells over-expressing DDX27 and cells carrying empty vector (EV) was determined by ANOVA with repeated-measures analysis of variances. **c** HCT116 cells stably overexpressing DDX27 and control cells were treated with 50 ng/ml TNF-α for 3 h. NFkB Signaling Pathway Plus PCR Array (Qiagen) which profiles the expression of 84 key genes related to NF-кB-mediated signal transduction was used to analyze the effect of DDX27 on the NF-кB signaling. The cutoff fold-change was set to 1.2. **d** and **e** SW480 or HCT116 cells were treated with 50 ng/ml TNF-α for indicated time. The mRNA expression levels of NF-кB target genes (BIRC3, CCL20, CXCL3, NFKBIA, TNF, and TNFAIP3) in SW480-EV and SW480-DDX27 (or HCT116-shCTL and HCT116-shDDX27) cells were measured by qPCR. Student’s *t*-test was performed between SW480-EV and SW480-DDX27 (or HCT116-shCTL and HCT116-shDDX27-2) cells at different time points. **f** SW480 or HCT116 cells stably overexpressing DDX27 and control cells were treated with 50 ng/ml TNF-α for indicated time. Isolated nucleus and cytoplasm were immunoblotted with indicated antibodies. Lamin A/C and α-tublin were used as nuclear marker and cytoplasmic marker, respectively. (*P < 0.05; **P < 0.01; ***P < 0.001)

To gain insights into the molecular mechanisms underlying pro-tumorigenic action of DDX27, we screened several important cancer pathways by luciferase reporter assays, including nuclear factor-кB (NF-кB), Wnt/β-catenin (TOPflash/FOPflash), STAT3 (APRE), p53, p38, p21, TGF-β and STAT5 (LHRE) signaling pathways. Among these signaling pathways, ectopic expression of DDX27 uniquely promoted NF-кB luciferase reporter activity in HCT116 cells (*P < *0.05; Fig. 5a). On the other hand, knockdown of DDX27 suppressed NF-кB signaling in both HCT116 and SW480 cells (both *P* < 0.05; Fig. 5a). In order to confirm whether the oncogenic function of DDX27 depends on NF-кB activation, CRC cells with or without ectopic expression of DDX27 were treated with two different NF-кB specific inhibitors, CAPE [16] and JSH-23 [17]. Treatment with either CAPE or JSH-23 markedly abolished the promoting effect of DDX27 on cell proliferation in HCT116 cells, which was further verified in SW480 cells, suggesting that DDX27 promotes CRC growth through activating NF-кB pathway **(**Fig. 5b). Consistent results were obtained by BrdU cell proliferation assay **(**Fig. S2A and S2B). NF-кB is a key regulator involved in tumor initiation, progression and metastasis [18]. Constitutive NF-кB activation is frequently observed in CRC [19, 20]. We next performed NF-кB PCR array, which profiles the expression of 84 key genes related to NF-кB-mediated signal transduction [21], to comprehensively analyze the effect of DDX27 on NF-кB signaling. As shown in Fig. 5c, 19 genes were up-regulated by ectopic expression of DDX27 in HCT116 cells treated with tumor necrosis factor alpha (TNF-α), while only three genes were down-regulated. Among these outlier genes, BIRC3, CCL20, CXCL3, NFKBIA, TNF, and TNFAIP3 are NF-кB target genes which were further validated to be significantly up-regulated by ectopic expression of DDX27 in HCT116 by qPCR (Fig. S2). Consistently, expression of these NF-кB target genes induced by TNF-α was enhanced in SW480 cells overexpressing DDX27 at different time points (Fig. 5d), whilst silencing of DDX27 in HCT116 cells reduced expression of the NF-кB target genes (Fig. 5e). To explore the mechanism by which DDX27 regulates NF-кB signaling, we examined the effect of DDX27 on activation of IκB kinase α (IKKα), degradation of IκBα and nuclear translocation of NF-kB p65. Upon TNF-α treatment for short time (≤1 h), phosphorylation of IKKα and p65, and degradation of IκBα in CRC cells with either DDX27 overexpression or silencing were similar to that of control (Fig. S3A-S3D), indicating that regulation of TNF-α-induced NF-kB signaling by DDX27 is independent of IKK. This was consistent with the observation that DDX27 did not interfere with p65 nuclear translocation upon TNF-α treatment at the initial time intervals (≤2 h) (Fig. 5f). Thus, we postulated that DDX27 may function downstream of IKK activation and IκBα degradation in simulating NF-кB signaling. Interestingly, TNF-α treatment over extended periods (3–5 h) resulted in decreased expression of IκBα, and increased nuclear accumulation of p65 specifically in CRC cells overexpressing DDX27 (Fig. 5f and S3E), implying a possible positive autocrine feedback loop between activation of NF-кB and translocation of NF-кB p65.

### DDX27 increases the interaction of nucleophosmin (NPM1) and NF-κB p65 to promote p65 DNA binding activity

**Fig. 6:**
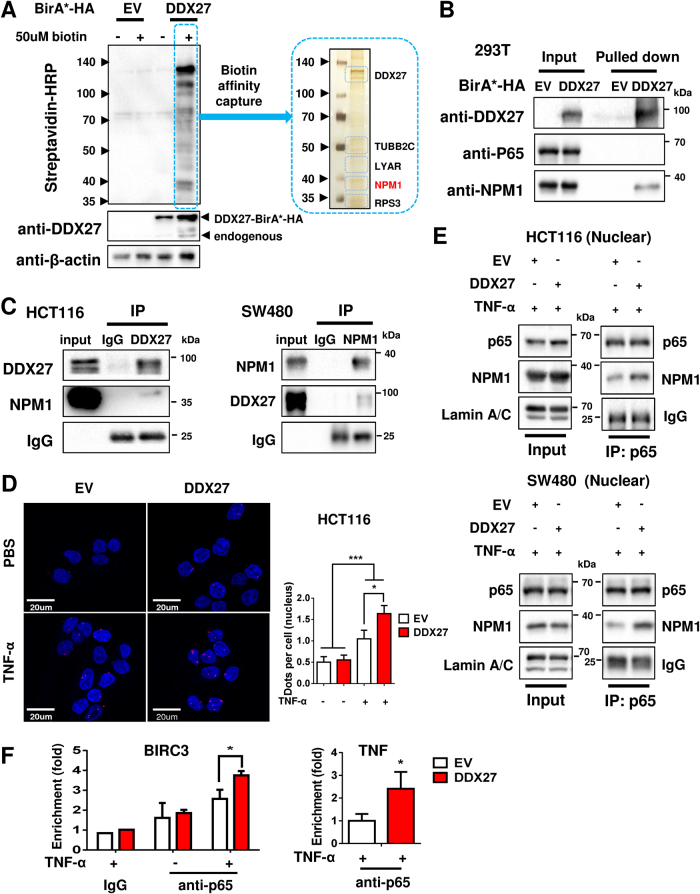
DDX27 promoted the interaction between NPM1 and p65 in nucleus and increased the binding activity of p65. **a** 293 T cells were transfected with pcDNA3.1-DDX27-BirA-HA or empty vector. Cells were incubated with 50 uM biotin and lysates were immunoblotted with indicated antibodies. 293 T cell lysates transfected with pcDNA3.1-DDX27-BirA-HA were pulled down by streptavidin beads and target proteins of DDX27 were identified by subsequent affinity purification mass spectrometry. **b** 293 T cell lysates transfected with pcDNA3.1-DDX27-BirA-HA were pulled down by streptavidin beads and immunoblotted with indicated antibodies. **c** The interactions between DDX27 and NPM1 were verified in HCT116 and SW480 with endogenous DDX27 immunoprecipitation and endogenous NPM1 immunoprecipitation, respectively. **d** In situ proximity ligation assay (PLA) detection of endogenous p65 and NPM1 interaction. HCT116 cells were incubated with or without TNF-α for 2 h. After fixation, in situ PLA for NPM1 and p65 was performed with NPM1 and p65-specific antibodies. The red dots (PLA signal) indicate an interaction. The nuclei were counterstained with DAPI (blue). The number of PLA dots in nucleus per cells was determined (at least 5 different regions (Obj: X63) were counted for each group). Mann–Whitney *U*-test was performed. **e** HCT116 and SW480 cells were treated with 50 ng/ml TNF-α for 2 h. In DDX27-overexpressing cells, enhanced protein interaction between NPM1 and NF-кB p65 was exhibited by endogenous p65 immunoprecipitation. **f** Recruitment of p65 to its target genes. HCT116 cells were incubated with or without TNF-α for 2 h, followed by ChIP assay. The precipitated DNA amounts containing the promoter regions of the genes (BIRC3 and TNF-α) by DDX27 overexpression were quantitatively analyzed by qPCR. Student’s *t*-test was performed. (*P < 0.05; ***P < 0.001)

**Fig. 7:**
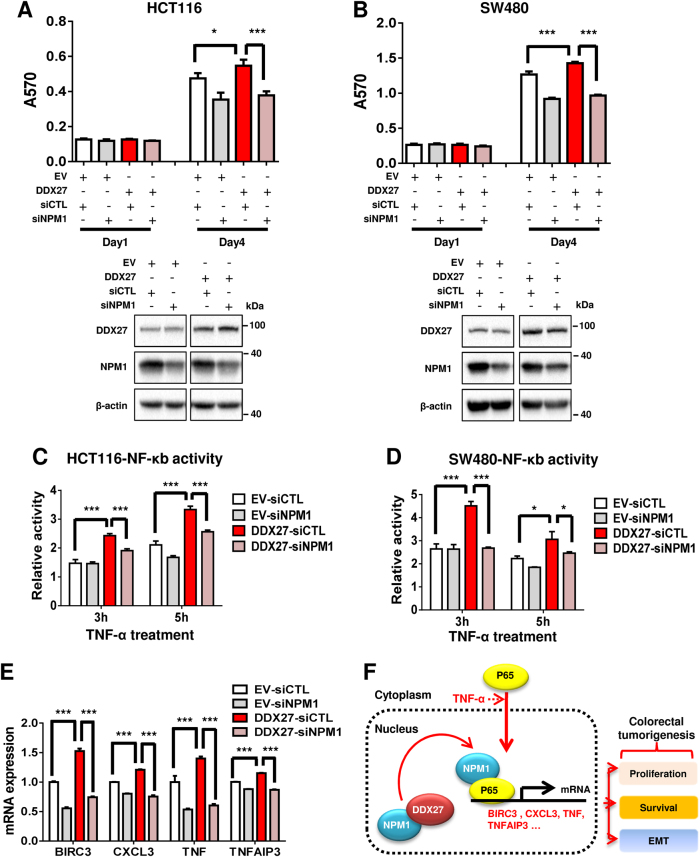
Oncogenic effect of DDX27 is dependent on NPM1-mediatd activation of NF-кB signaling. **a** and **b** Transfection with control-siRNA (siCTL) or specific NPM1-siRNA (siNPM1) in the control and DDX27-overexpressing HCT116 (or SW480 cells) revealed that silencing of NPM1 abolished growth advantage conferred by ectopic expression of DDX27. The cell lysates were blotted with indicated antibody. **c** and **d** HCT116 or SW480 cells were treated with 50 ng/ml TNF-α for indicated time. Silencing of NPM1 abolished DDX27-enhaced activation of NF-кB luciferase activity. **e** HCT116 cells were treated with 50 ng/ml TNF-α for 3 h. The mRNA expression of NF-кB target genes (BIRC3, CXCL3, TNF, and TNFAIP3) were measured by qPCR. **f** Proposed mechanistic scheme of DDX27. DDX27 directly interacts with NPM1 and promotes its interaction with p65 in the nucleus, hence increased the binding activity of p65 to promoters of NF-кB target genes and enhanced the transcription. DDX27 likely mediates a positive autocrine feedback on NF-κB. Enhanced NF-кB signaling will increase cell proliferation, inhibit apoptosis and promote metastasis for colorectal tumorigenesis. (*P < 0.05; **P < 0.01; ***P < 0.001)

Recent reports have suggested that members of the DEAD box family generally act as components of multi-protein complexes and their functions are likely to be affected by their interacting partners [12]. To identify candidate proteins interacting with DDX27, we expressed HA-tagged DDX27 fused to N-terminus of biotin ligase BirA in 293T cells, and performed affinity purification mass spectrometry using streptavidin beads (Fig. 6a). Among the biotinylated protein targets identified, NPM1 was the most abundant protein (Fig. 6a). Existence of NPM1 in these biotinylated protein targets was further confirmed using anti-NPM1 antibody (Fig. 6b). On the other hand, NF-кB p65 was not identified (Fig. 6b), implying no direct interaction between DDX27 and p65. NPM1 is frequently overexpressed in cancer and is mainly located in the nucleus [22]. The interaction of DDX27 and NPM1 in the nucleus was demonstrated by endogenous reciprocal co-immunoprecipitation (Co-IP) assay in both HCT116 and SW480 cells (Fig. 6c). However, DDX27 regulated neither expression nor nuclear translocation of NPM1 **(**Fig. S4A**)**. It is reported that NPM1 is associated with nuclear NF-κB p65 to enhance the DNA binding activity of NF-κB in response to TNF-α stimulation, and that NPM1 and NF-κB are likely to act cooperatively to regulate tumor progression [23]. It is therefore plausible that DDX27 may regulate the interaction of NPM1 and p65 in the nucleus. Proximity ligation assay (PLA) was performed to visualize interactions of NPM1 and p65. As shown in Fig. 6d, TNF-α treatment significantly increased endogenous interaction of NPM1 with p65 in the nucleus (*P < *0.001). Moreover, in response to TNF-α stimulation, the number of nuclear PLA dots per cell was significantly increased (∼1.5 fold) in HCT116 cells with ectopic expression of DDX27 as compared to controls, indicating that up-regulation of DDX27 significantly enhanced the interaction of NPM1 with p65 in nucleus. Consistent result was obtained by endogenous IP assay in HCT116 and SW480 cells (Fig. 6e). TNF-α did not regulate gene expression **(**Fig. S4A**)**, nuclear translocation **(**Fig. S4A**)**, or interaction of DDX7 and NPM1 in CRC cells (Fig. S4B**)**. Consequently, the binding activity of p65 to target gene promoters (BIRC3 and TNF) was found to be significantly enhanced in HCT116 cells overexpressing DDX27 by chromatin immunoprecipitation assay (Fig. 6f). We sought to determine whether NPM1 is critical to the oncogenic effect of DDX27. Knockdown of NPM1 abrogated the tumor-promoting function of DDX27, as indicated by MTT assays, without affecting DDX27 expression **(**Figs. 7a, b**)**. Moreover, NPM1 knockdown abolished DDX27-mediated NF-κB signaling activation and the expression of NF-κB target genes, as evidenced by luciferase reporter assays and qPCR, respectively (Figs. 7c-e). Collectively, these results suggest that DDX27 promoted the interaction between NPM1 and p65 in the nucleus, thereby increasing the DNA binding activity of p65 and promoting NF-κB signaling (Fig. 7f).

## Discussion

In this study, our comprehensive analysis of TCGA-CRC cohort led to the novel identification of DDX27 as a putative oncogene with extremely high frequency of copy number gain in CRC, an observation independently verified in our CRC cohort. DDX27 is located on chromosome 20q13.13. DNA copy number gains of chromosomes 20q are commonly found in CRC and are associated with colorectal carcinogenesis [24]. DNA copy number gain is an important cause that drives aberrant overexpression of oncogenes in cancer. We found that DDX27 expression is significantly up-regulated in human CRC at mRNA level and protein level. DDX27 mRNA overexpression was positively correlated with DNA copy number gain inferring that gain of DNA copy number contributes to DDX27 up-regulation in CRC.

DDX27 belongs to the DEAD box nucleic acid helicase family which shares a conserved “DEAD-box” sequence motif [11]. Here, we reported for the first time that DDX27 exerted oncogenic role in CRC. DDX27 promoted cell proliferation in CRC cells, an effect that primarily involves suppression of apoptosis as exemplified by the inhibition of caspase cascade. In accordance with in vitro data, DDX27 promoted tumor growth in mouse xenograft models; whilst its knockdown markedly suppressed xenograft growth. Apart from promoting CRC cell growth, DDX27 also regulated metastatic ability by promoting cell migration and invasion in vitro and lung metastasis in vivo. DDX27 positively regulated EMT in CRC cells through up-regulation of Vimentin and Slug, while simultaneously down-regulating E-cadherin expression. Vimentin and Slug are mesenchymal-related proteins that promote migratory and invasive properties of carcinoma cells [25, 26]. Meanwhile, E-cadherin loss is associated with increased cell motility [27]. Collectively, these results indicate that DDX27 exerts oncogenic properties in colorectal carcinogenesis via promoting cell proliferation, inhibiting apoptosis and increasing metastatic abilities. In agreement with our results, recent studies have suggested that several DEAD box proteins have multifunctional roles in biological processes associated with tumorigenesis [13–15]. Among them, DDX1 is frequently co-amplified with N-myc in several types of cancers [28, 29] and plays a crucial role in testicular tumorigenesis [13]. Hence, the frequent gain of copy number of DDX27 in CRC, together with its remarkable oncogenic effect in CRC cells in vitro and in vivo suggests that DDX27 play an important role in triggering CRC initiation and progression.

We examined the molecular mechanism of DDX27 acting as an oncogenic factor in CRC and identified NF-κB pathway as the major target of DDX27 in CRC. NF-кB transcription factor family regulates a mass of genes widely involved in inflammation, angiogenesis, cell proliferation, and metastasis [18]. Constitutive activation of NF-кB pathway is commonly observed in CRC, leading to tumor initiation, invasion and drug resistance [19, 20]. NF-кB activity is induced by various stimuli such as reactive oxygen species, TNF-α, interleukin 1β, and lipopolysaccharides [18]. Upon stimulation, IKK catalyzes phosphorylation and degradation of IκBα. Degradation of IκBα contributes to the release of NF-кB and allows its localization in the nucleus to initiate gene transcription [30]. DDX27 confers NF-κB-mediated growth advantage to CRC cells, which can be abolished by NF-кB specific inhibitors. DDX27 potentiated a prolonged NF-кB response without affecting its initial activation upon TNF-α simulation, implying that it functions downstream of IKK activation and IκBα degradation. DDX27 likely mediates a positive autocrine feedback on NF-κB, leading to enhanced accumulation of p65 in the nucleus after extended TNF-α simulation for 3 h. As a consequence, DDX27 substantially enhanced the expression of NF-kB target genes (BIRC3, CCL20, CXCL3, NFKBIA, TNF, and TNFAIP3) induced by TNF-α. NF-κB target genes are documented to play diverse roles in promoting cell survival and metastasis. BIRC3 (c-IAP2) has been shown to exert positive feedback control on NF-κB via targeting IκBα for degradation and facilitate cell survival by suppressing caspase-8 activation [31–33]. CCL20 and CXCL8 co-expression was shown to be negatively correlated with E-cadherin expression and they cooperatively promote cell metastasis via inducing EMT [34]. On the other hand, exogenous CXCL3 enhanced migration of prostate cancer cells [35]. In this connection, DDX27 may promote tumor survival and EMT via activation of NF-κB. In line with our findings, DDX1 acted as a co-activator of NF-кB to enhance NF-кB-mediated transcription [36]. DDX5 promoted cancer cell viability via direct regulation of NF-кB p50 [37]. Collectively, our work unveiled a novel DEAD Box protein that promotes NF-кB signaling in cancer.

Emerging evidence indicate that DDX proteins regulate signaling pathways as part of multi-protein complexes. Here, we established proximity-dependent biotinylation system [38] and identified NPM1 as the interacting protein of DDX27, which was independently verified by endogenous Co-IP. NPM1 was reported to interact with NF-κB in the nucleus to stimulate the binding of NF-κB to target gene promoters in response to TNF [23]. Accordingly, ectopic expression of DDX27 in CRC cells significantly increased the interaction of NPM1 and p65 upon TNF-α treatment, following by increased binding of NF-κB to promoters of NF-κB target Genes. Moreover, NPM1 knockdown abrogated DDX27-mediated NF-κB signaling and CRC cell growth. Hence, DDX27- NPM1-NF-κB forms a functional axis that cooperatively regulates tumor progression.

Finally, the clinical implication of DDX27 was assessed in three independent CRC cohorts, which demonstrated that high expression of DDX27 predicts adverse prognosis in CRC patients, consistent with its putative pro-tumorigenic role in CRC. It is worth noting that prognostic significance of DDX27 might be contributed by the CIN phenotype considering that DDX27 is located on chromosome 20q, where frequent DNA copy number occurs in CRC. It will be of interest if DDX27 may serve as a surrogate marker for CIN in future studies.

In summary, we demonstrated for the first time that DDX27 copy number gain is a common event in CRC which leads to DDX27 overexpression. DDX27 is a novel oncogene involved in colorectal carcinogenesis through increasing cell proliferation, inhibiting apoptosis and promoting metastasis. The oncogenic function of DDX27 was mediated by promoting NF-κB signaling through direct interaction with NPM1. High DDX27 predicts poor prognosis for CRC patients.

## Materials and methods

### Human CRC samples

Three cohorts of CRC patients were included. For TCGA cohort, CNA and mRNA expression data across 24776 genes of 615 CRC samples were acquired from UCSC Cancer Browser (https://genome-cancer.ucsc.edu/). Surgically excised CRC tissues and surrounding non-tumor tissues were obtained from 199 patients (199 CRC and 151 adjacent normal tissues) with CRC from the Beijing University Cancer Hospital (Beijing cohort) and 278 patients (278 CRC and 252 adjacent normal tissues) with colon cancer from Ren Ji Hospital (Shanghai cohort). The clinicopathological features of patients were shown in Table S4 and S5. Informed consent was provided and patients were regularly followed up. In Beijing cohort, DNA and RNA were isolated from the same CRC tissues of 103 randomly selected patients. In Shanghai cohort, 18 patients were excluded from survival analysis because of incomplete follow-up information. Relapse-free survival and overall survival information were available for Beijing and Shanghai cohort, respectively. This study was approved by the ethics committee of the Chinese University of Hong Kong, Beijing University Cancer Hospital and Ren Ji Hospital.

### Tissue microarray assay

Tissue microarrays were stained with DDX27 antibody (Sigma-Aldrich, St. Louis, MO, USA) according to the procedure mentioned previously [39]. The positive percentage was scored as follows: 0, no positive staining; 1, in between 1 and 25% cells; 2, in between 26 and 50% cells; 3, in between 51 and 75% cells; 4, in more than 75% cells. The staining intensity was scored as follows: 0, negative; 1, weak; 2, moderate; and 3, high intensity. The immunoreactive score in cytoplasm and nucleus was calculated as staining intensity score x percentage of DDX27-positive cells. The staining score of DDX27 was determined as sum of cytoplasmic score plus nucleic score. The results were scored independently by two pathologists.

### Western blot analysis and endogenous immunoprecipitation

Western blot analysis was performed as previously described [40]. The primary antibodies used were NPM1 (Abcam, Cambridge, UK); Caspase 8, Cleaved Caspase 8,Caspase 3, Cleaved Caspase 3, PARP, Cleaved PARP, E-Cadherin (4A2), Slug, IKBα, phospho NF-κB p65(Ser536), NF-κB p65 (D14E12), Phospho-IKKα/β (Ser176/180), IKKα, alpha Tubulin, LaminA/C (Cell Signaling Technology, Danvers, MA, USA); Vimentin(ImmunoWay Biotechnology Company, TX USA); β-actin (Santa Cruz Biotechnology); DDX27 (Sigma-Aldrich, St. Louis, MO, USA). For endogenous immunoprecipitation, immune complexes were precipitated by anti-DDX27, anti-NPM1 or anti-p65 and analyzed by western blot.

### BioID proximity-dependent biotinylation

The experiment was performed following the protocol provided by Kyle J. Roux [38]. DDX27 cDNA was fused into pcDNA3.1 MCS-BirA(R118G)-HA (addgene, Cambridge, MA, USA) and 293 T cells were transfected with pcDNA3.1 -DDX27-BirA-HA or empty vector. 48 h after transfection, 50 μM biotin was added to cells for 24 h. After washed with PBS, cells were lysed in RIPA buffer (25 mM Tris HCl, 150 mM NaCl, 1.0% NP-40, 1.0 mM EDTA and 5% Glycerol) supplemented with with 1 × complete protease inhibitor (Roche Applied Sciences). The biotinylated proteins were purified using Dynabeads® Streptavidin (Thermo Fisher Scientific, Waltham, MA, USA) and eluted in SDS-PAGE loading buffer. Eluted proteins were fractionated by SDS-PAGE and visualized by silver staining. Candidate targets were excised from gels and subjected to in-gel digestion and matrix-assisted laser desorption/ionization time-of-flight/ time-of-flight (MALDI-TOF/TOF) mass spectrometry.

### Statistical analysis

All results were expressed as mean ± SD unless otherwise indicated. To compare the difference between two groups, Mann-Whitney U-test, Wilcoxon matched-pairs test and Student’s *t*-test were performed. The difference between growth rates was determined by ANOVA with repeated-measures analysis of variances. The correlation between DDX27 copy number and mRNA expression was analyzed using Spearman rank correlation. The Pearson chi-square test or Fisher’s exact test was used for analysis of the associations between patient clinicopathological characteristics and DDX27 expression. Kaplan-Meier analysis and log-rank test were performed to evaluate the association between DDX27 expression and patient survival. Cutoff value of DDX27 in Beijing or TCGA cohorts was analyzed by survival significance analysis using the tool Cutoff Finder (http://molpath.charite.de/cutoff/) [41]. COX proportion hazard regression model was performed to assess the prognostic value of DDX27 expression. All statistical tests were performed using Graphpad Prism 5.0 (GraphPad Software Inc., San Diego, CA, USA) or SPSS, version 20.0 (SPSS Inc, Chicago, IL), and a two-tailed P-value of 0.05 was considered statistically significant.

## Electronic supplementary material

Supplementary Methods

Supplementary Figure 1-re

Supplementary Figure 2-re

Supplementary Figure 3

Supplementary Figure 4

Supplementary Tables 1-13
