# Supplementary material for: DEAD-box helicase 27 promotes colorectal cancer growth and metastasis and predicts poor survival in CRC patients
Source: Oncogene. 2018 Mar 14;37(22):3006–21. doi: 10.1038/s41388-018-0196-1 (PMC5978808; doi:10.1038/s41388-018-0196-1)
Supplement: Supplementary file 1 — Supplementary Methods [file 41388_2018_196_MOESM1_ESM.docx]

**Supplementary Methods**

**PCR Copy Number Analysis**

DNA was extracted from 103 frozen CRC samples (randomly selected) of Beijing Cohort using the AllPrep DNA/RNA/Protein Kit (QIAGEN, Hilden, Germany). The purified genomic DNA was amplified with a probe specific to target gene DDX27 (Hs01275908_cn) by TaqMan Copy Number Assays (Applied Biosystems, Waltham, MA) in real-time polymerase chain reaction, according to the protocol provided by company. DDX27 copy number was analyzed using CopyCaller software v2.0 (Applied Biosystems).

**Real-time Quantitative PCR**

RNA was extracted using Trizol reagent (Invitrogen, Carlsbad, CA, USA). Complementary DNA (cDNA) was synthesized using Transcriptor Reverse Transcriptase (Roche Applied Sciences, Indianapolis, IN, USA). Quantitative PCR was performed on a LightCycler 480 real-time PCR system (Roche Applied Science) using the LightCycler 480 SYBR Green I Master (Roche Applied Science). The primers used were listed in **Table S13**. The relative expression level was normalized to β-actin and corrected for the mean of the abundance of control group.

**Cell Lines**

Human CRC cell lines (DLD-1, LoVo, HT29, HCT116, SW1116, SW480, and SW620) and HEK293T were obtained from the American Type Culture Collection (ATCC, Manassas, VA, USA). The colon normal epithelial cell line NCM460 was obtained from Incell Corporation (San Antonio, Texas, USA). HT29 and HCT116 were cultured in McCoy’s 5A modified medium (Invitrogen). LoVo, SW480, SW620, NCM460 and HEK293T were cultured in DMEM (Invitrogen). DLD-1 and SW1116 were cultured in 1640 medium (Gibco, Rockville, MD, USA). All medium were supplemented with 10% fetal bovine serum (Gibco).

**Lentivirus Packaging and Transduction**

Two short hairpin RNAs targeting human DDX27 (shDDX27-1: 5’- GCUGAUUUCAACCCUGAUUTT-3’ and shDDX27-2: 5’- GCCCAGAUCAAUACAGCAATT- 3’) and a non-targeting RNA sequence serving as a negative control were cloned into the pGPU6 vector (Genepharma, Shanghai, China). Virus packaging was performed in HEK293T cells.

The full-length human DDX27 cDNA (NM_017895.7) was cloned into the pCMV vector (Genepharma). Virus packaging and transduction followed the same steps described above. Empty vector (EV) was served as control.

**Small Interfering RNA-Mediated Gene Silencing**

The sequences of NPM1-small interfering RNAs (Genepharma) are as follows: GUGCAAAGGAUGAGUUGCATT (sense: 5’–3’) and UGCAACUCAUCCUUUGCACTT (anti-sense: 5’–3’). A non-targeting RNA sequence was served as a negative control.

**Cell Viability and Colony Formation Assay**

Cells (1 × 10^3^ per well) were seeded in a 96-well plate. MTT (5 mg/ml; Promega, Madison, WI, USA) was added to the culture to equal one-tenth the original culture volume. The reaction was stopped by 100uL/well dimethyl sulfoxide (DMSO). Absorbance (A570) was measured. For colony formation assay, cells (500 per well) were seeded in 12-well plate for 2 weeks. Colonies were fixed with methanol/acetone (1:1), stained with Gentian violet, and counted. Caffeic acid phenethyl ester (CAPE) and 4-methyl-1-N- (3-phenylpropyl) benzene-1,2-diamine (JSH-23) were purchased from ApexBio company (Shanghai, China).

**BrdU cell proliferation assay**

The BrdU cell proliferation assay kit was obtained from Cell Signaling Technology (Danvers, MA, USA). Cells were plated in 96-well plates at 2×10^3^ cells/well and incubated overnight. CAPE and JSH-23 at indicated concentration was added to the culture for 24h. Later BrdU was added in each well and incubated for 3h, followed by fixation and wash. The BrdU detection antibody and HRP-conjugated secondary antibody were used successively. TMB substrate was added to the plate for reaction and absorbance at 450 nm was measured.

**Apoptosis Assay**

Staurosporine (STS) (Sigma, St. Louis, MO) was used to induce apoptosis. HCT116 and SW480 cells were treated with 1.0 uM STS and 1.5 uM STS for three hours, respectively. Apoptosis was determined using the Annexin-phycoerythrin/7-aminoactinomycin D staining kit (BD Biosciences, San Jose, CA).

**Wound-Healing and Invasion Assays**

Confluent cultures in 6-well plates were scratched with sterile tips, washed with PBS and cultured in 1% FBS supplemented culture medium. Cells were photographed at 0, 24 and 48 hours. For invasion assays, Matrigel-coated chambers (Becton Dickinson, Waltham, MA, USA) were used. Briefly, 1×10^4^ cells were seeded into the upper chamber in serum-free culture medium. The lower chamber was filled with completed medium with 10% FBS. After 48h, cells that have invaded through the membrane were stained with crystal violet and counted.

**Subcutaneous Xenograft and Experimental Metastasis Mouse Models**

For subcutaneous xenografts, SW480-EV and SW480-DDX27 (or HCT116-shCTL cells and HCT116-shDDX27) cells were injected subcutaneously into the left and right dorsal flank of 4-week-old male Balb/c nude mice (2×10^6^ cells in 0.1 mL phosphate-buffered saline (PBS) /mice), respectively. Tumor size was measured every 2 days intervals using a digital caliper. Tumour volume (mm^3^) was calculated as follows: volume= (shortest diameter) ^2^× (longest diameter) ×0.5. At the end point, tumors were harvested and weighed. For metastasis model, stable HCT116-shCTL cells and HCT116-shDDX27 (3×10^6^ cells in 0.1 mL PBS) were injected intravenously via the tail vein. After 6 weeks, the mice were sacrificed. Lungs were harvested, sectioned and stained with H&E staining. All animal experimental procedures were approved by the Animal Ethics Committee of the Chinese University of Hong Kong.

**Luciferase Reporter Assay**

Cancer pathway reporters including NF-кB, Wnt/β-catenin (TOPFlash and FOPFlash), STAT3 (APRE), p53, p38, p21, TGF-β, and LHRE (STAT5) were included in this study. Briefly, cells were seeded into a 24-well plate and transfected with the firefly (cancer pathway reporter) and renilla (internal control) luciferase reporters. Two days after transfection, cells were harvested and the firefly and renilla luminescence were measured by the dual-luciferase reporter assay system (Promega). Reporter activity was dertermined as the ratio of firefly to Renilla luciferase activity.

**Chromatin Immunoprecipitation Assay**

According to the Magna ChIP™ protocol (Merck Millipore, ‎Billerica, Mass.‎, USA), cells were fixed with 1% formaldehyde for crosslinking at room temperature for 10 minutes. Glycine was added to cells to quench unreacted formaldehyde for 5 minutes. Cell pellets in SDS Lysis Buffer containing 1X Protease Inhibitor Cocktail were sonicated at 4ºC to yield fragments from 100 to 500 bps. Immunoselection was performed using anti-NF-κB p65 (D14E12) antibody and ChIP-grade Protein A/G Magnetic Beads (Merck Millipore). Protein-DNA crosslinks were reversed at 65°C and DNA is purified for qPCR. The primers used were listed in **Table S13**.

**Immunofluorescence**

Cells were grown on coverslips, fixed with 4% paraformaldehyde and permeabilized with 0.2% Triton X-100, blocked in 5% bovine serum albumin in PBS. Cells were incubated with anti-DDX27 antibody (Sigma-Aldrich, St. Louis, MO, USA) overnight at 4°C, followed by anti-rabbit IgG secondary antibody conjugated with Alexa Fluor 594 (Yeasen, Shanghai, China) in the dark for 1 h, and Phalloidin-iFluor 647 reagent (Abcam, Cambridge, UK) for 20 minutes. Nuclei were counterstained with DAPI (Life technologies, Carlsbad, CA).

***In Situ* Proximity Ligation Assay**

*In situ* interactions of NPM1 and p65 were detected using the Duolink *In Situ* proximity ligation assays (PLA) detection kit (Sigma-Aldrich) containing the PLA probe anti-rabbit minus, PLA probe anti-mouse plus and Detection Kit Red. The assay was performed following the manufacturer's instructions.
