# Supplementary figures and images for: DEAD-box helicase 27 promotes colorectal cancer growth and metastasis and predicts poor survival in CRC patients
Source: Oncogene. 2018 Mar 14;37(22):3006–21. doi: 10.1038/s41388-018-0196-1 (PMC5978808; doi:10.1038/s41388-018-0196-1)

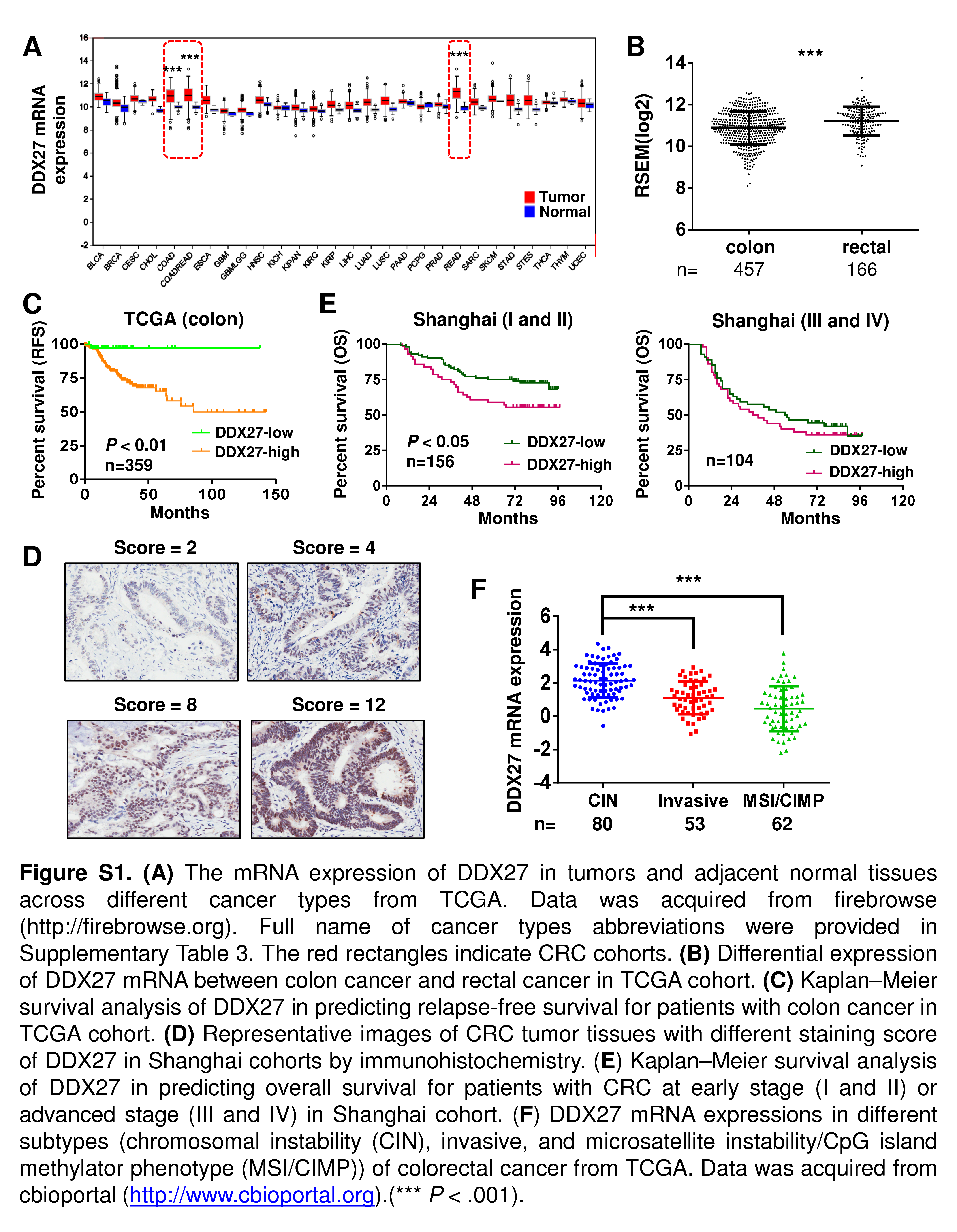

Supplement: Supplementary file 2 — Supplementary Figure 1-re [file 41388_2018_196_MOESM2_ESM.tif]

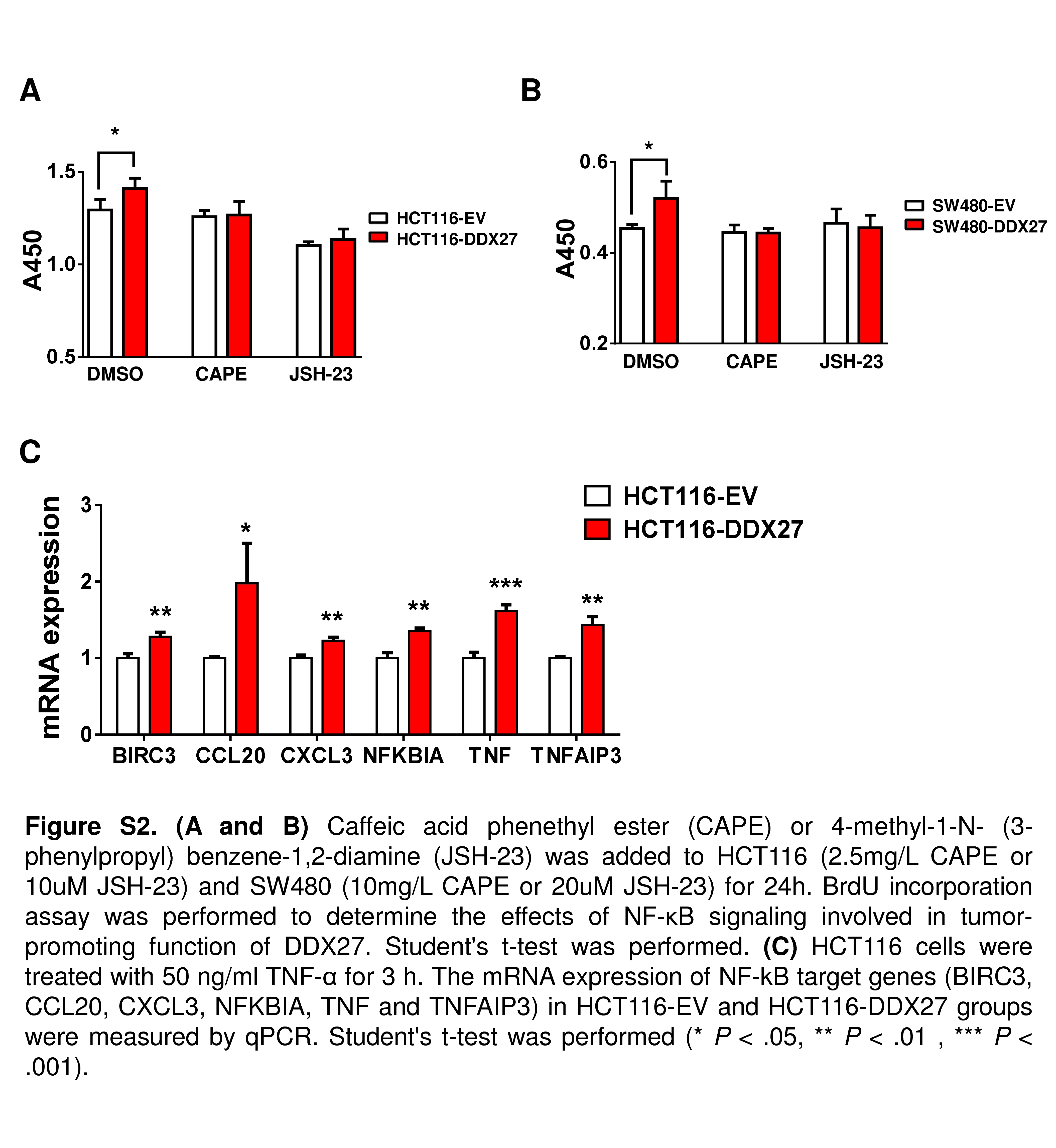

Supplement: Supplementary file 3 — Supplementary Figure 2-re [file 41388_2018_196_MOESM3_ESM.tif]

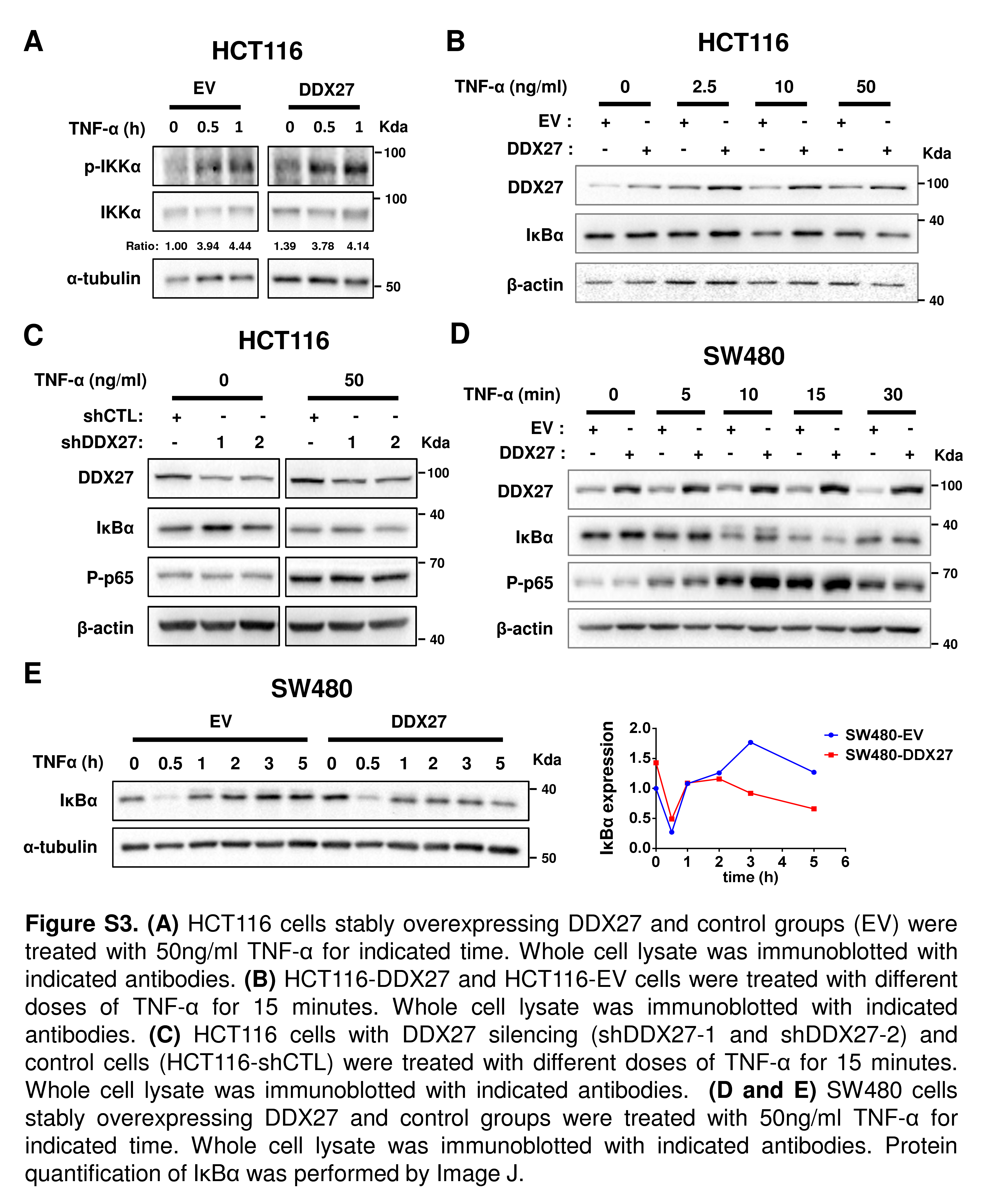

Supplement: Supplementary file 4 — Supplementary Figure 3 [file 41388_2018_196_MOESM4_ESM.tif]

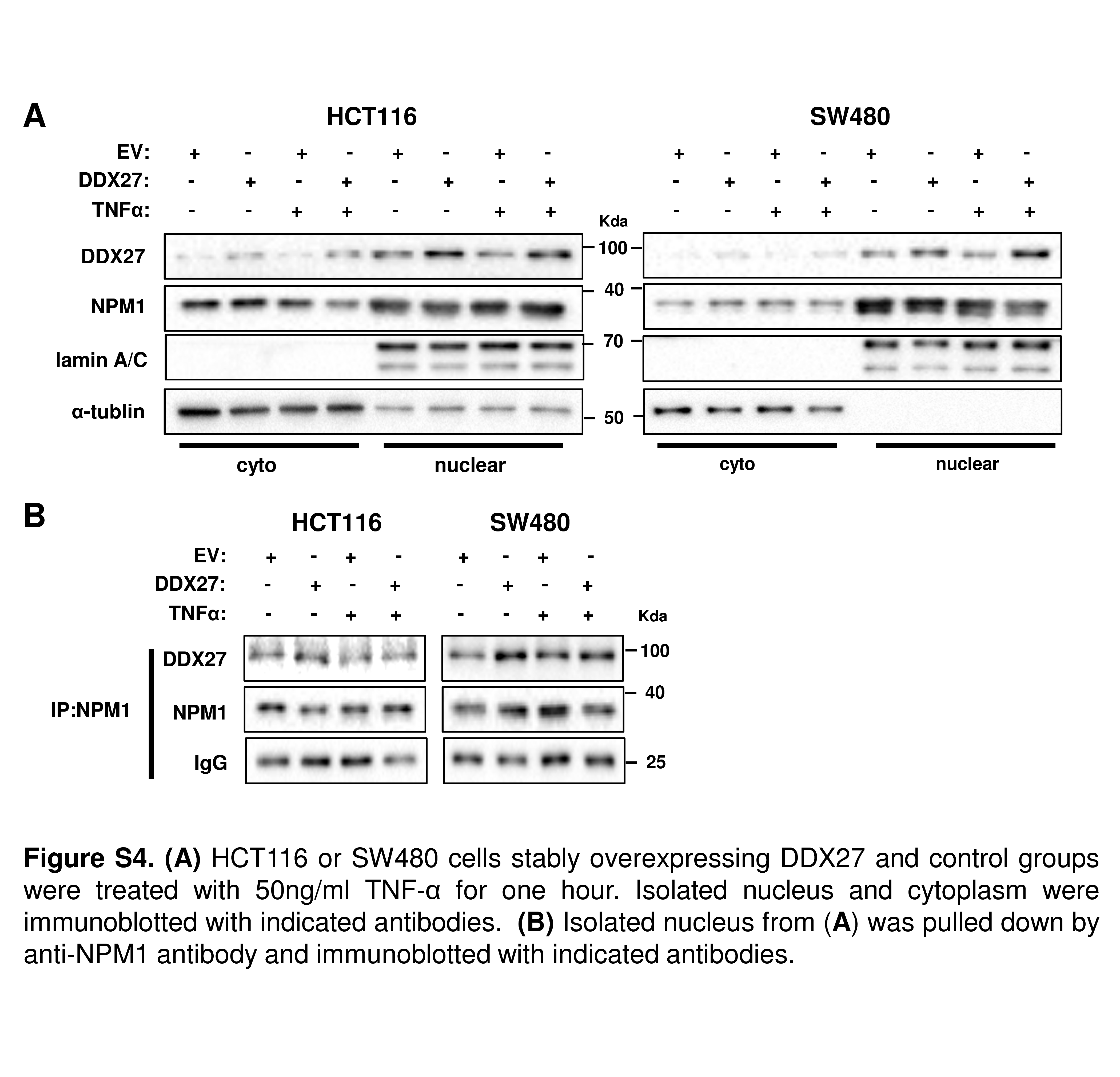

Supplement: Supplementary file 5 — Supplementary Figure 4 [file 41388_2018_196_MOESM5_ESM.tif]
