# Supplementary material for: DEAD-box helicase 27 promotes colorectal cancer growth and metastasis and predicts poor survival in CRC patients
Source: Oncogene. 2018 Mar 14;37(22):3006–21. doi: 10.1038/s41388-018-0196-1 (PMC5978808; doi:10.1038/s41388-018-0196-1)
Supplement: Supplementary file 6 — Supplementary Tables 1-13 [file 41388_2018_196_MOESM6_ESM.pdf]

Supplementary Table 1: Top ranked genes with the highest frequency of copy number gain in 615 human CRC from TCGA

Gene	Lost	Neutral	Gained	Percentage
ARFGEF2	0	141	474	0.771
CSE1L	0	141	474	0.771
**DDX27**	**0**	**141**	**474**	**0.771**
EYA2	0	141	474	0.771
FAM65C	0	141	474	0.771
LINC00494	0	141	474	0.771
MIR3616	0	141	474	0.771
MIR645	0	141	474	0.771
NCOA3	0	141	474	0.771
PREX1	0	141	474	0.771
PTPN1	0	141	474	0.771
RN7SKP33	0	141	474	0.771
RN7SL243P	0	141	474	0.771
RN7SL636P	0	141	474	0.771
RN7SL672P	0	141	474	0.771
SNORD12	0	141	474	0.771
SNORD12B	0	141	474	0.771
SNORD12C	0	141	474	0.771
STAU1	0	141	474	0.771
SULF2	0	141	474	0.771
ZFAS1	0	141	474	0.771
ZMYND8	0	141	474	0.771
ZNFX1	0	141	474	0.771

Supplementary Table 2: Somatic mutations of DDX27 detected by whole-exome sequencing in TCGA CRC cohorts

Sample ID	Cancer Type	AA change	Type	Copy #	COSMIC	MS	VS	Mutation Assessor	Center	Chr	Start Pos	End Pos
TCGA-AA-3877-01	Colorectal Cancer	K691Rfs*4	FS del	Diploid		S	U		Broad	chr20	47858504	47858504
TCGA-AA-3966-01	Colorectal Cancer	K691Rfs*4	FS del	Gain		S	U		Broad	chr20	47858504	47858504
TCGA-AA-3715-01	Colorectal Cancer	R562H	Missense	Diploid	1	S	V	Low	Broad	chr20	47852952	47852952
TCGA-AA-A01V-01	Colorectal Cancer	K474R	Missense	Diploid	1	S	V	Neutral	Broad	chr20	47851526	47851526
TCGA-AA-A022-01	Colorectal Cancer	V292M	Missense	Diploid	1	S	V	Medium	Broad	chr20	47845326	47845326
TCGA-AG-3901-01	Colorectal Cancer	R502W	Missense	Gain	1	S	V	High	Broad	chr20	47851609	47851609

Supplementary Table 3: Differential expression analysis of DDX27 across various cancer types in TCGA datasets

Cancer type	Fold change (Tumor vs. Normal)	Cancer type	Fold change (Tumor vs. Normal)
**READ**	**2.49**	BLCA	1.24
**COADREAD**	**2.07**	KIPAN	1.18
CHOL	1.99	PRAD	1.17
**COAD**	**1.99**	GBMLGG	1.16
ESCA	1.76	KIRC	1.16
STES	1.73	SKCM	1.13
LUSC	1.71	CESC	1.12
STAD	1.71	GBM	1.10
LUAD	1.56	PAAD	1.10
SARC	1.42	THYM	1.10
LIHC	1.35	THCA	1.06
KIRP	1.34	UCEC	1.06
HNSC	1.33	KICH	1.00
BRCA	1.28	PCPG	0.83

BLCA: Bladder urothelial carcinoma; **BRCA**: Breast invasive carcinoma; **CESC**: Cervical and endocervical cancers; **CHOL**:Cholangiocarcinoma; **COAD**:Colon adenocarcinoma; **COADREAD**: Colorectal adenocarcinoma; **ESCA**: Esophageal carcinoma; **GBM**: Glioblastoma multiforme; **GBMLGG**: Glioma; **HNSC**: Head and Neck squamous cell carcinoma; **KICH**: Kidney Chromophobe; **KIPAN**: Pan-kidney cohort (KICH+KIRC+KIRP); **KIRC**: Kidney renal clear cell carcinoma; **KIRP**: Kidney renal papillary cell carcinoma; **LIHC**: Liver hepatocellular carcinoma; **LUAD**: Lung adenocarcinoma; **LUSC**: Lung squamous cell carcinoma; **PAAD**: Pancreatic adenocarcinoma; **PCPG**: Pheochromocytoma and Paraganglioma; **PRAD**: Prostate adenocarcinoma; **READ**: Rectum adenocarcinoma; **SARC**: Sarcoma; **SKCM**: Skin Cutaneous Melanoma; **STAD**: Stomach adenocarcinoma; **STES**: Stomach and Esophageal carcinoma; **THCA**: Thyroid carcinoma; **THYM**: Thymoma; **UCEC**: Uterine Corpus Endometrial Carcinoma.

Supplementary Table 4: Clinicopathologic Features of DDX27 Expression in Colorectal Cancer from Beijing.

Beijing cohort Variable	Low DDX27 expression (n=47)	High DDX27 expression (n=152)	*P* value
**Age**, mean ± SD	57.5 ± 14.3	59.2 ± 12.4	0.421
**Sex**			
Male	28 (59.6%)	94 (61.8%)	0.864
Female	19 (40.4%)	58 (38.2%)	
**Location**			
Colon	35 (74.5%)	66 (43.4%)	<0.001
Rectum	12 (25.5%)	86 (56.6%)	
**TNM stage**			
I, II	22 (46.8%)	70 (46.0%)	1.000
III, IV	25 (53.2%)	82 (54.0%)	

NOTE. Values are n (%) unless otherwise indicated.

Supplementary Table 5: Clinicopathologic Features of DDX27 Expression in Colorectal Cancer from Shanghai cohorts

Shanghai cohort Variable	Low DDX27 expression (n=154)	High DDX27 expression (n=106)	*P* value
**Age**, mean ± SD	66.9 ± 11.5	67.1 ± 10.6	0.888
**Sex**			
Male	75 (48.7%)	66 (62.3%)	0.032
Female	79 (51.3%)	40 (37.7%)	
**TNM stage**			
I, II	100 (64.9%)	56 (52.8%)	0.054
III, IV	54 (35.1%)	50 (47.2%)	

NOTE. Values are n (%) unless otherwise indicated.

Supplementary Table 6: Cox regression analysis for Relapse -Free Survival in CRC patients (Beijing cohort)

**Beijing cohort (n=199)**	Univariate Analysis		Multivariate Analysis	
	Hazard ratio (95% CI)	P value	Hazard ratio (95% CI)	P value
DDX27 expression (high vs. low)	2.482 (1.059-5.819)	0.037	2.667 (1.116-6.374)	0.027
Age (>60 vs. <=60)	1.118 (0.645-1.938)	0.691	1.155 (0.663-2.014)	0.611
Male vs. female	1.314 (0.727-2.374)	0.366	1.329 (0.731-2.418)	0.351
Localisation (Colon vs. Rectum)	1.139 (0.657-1.973)	0.644	1.341 (0.764-2.354)	0.307
Stage (III, IV vs.I, II)	2.208 (1.221-3.993)	0.009	2.146 (1.185-3.885)	0.012

Supplementary Table 7: Clinicopathologic Features of DDX27 Expression in Colorectal Cancer (colon) from Beijing.

**Beijing cohort (colon) Variable**	**Low DDX27 expression (n=35)**	**High DDX27 expression (n=66)**	***P* value**
**Age**, mean ± SD	59.6 ± 13.7	59.7 ± 12.5	0.971
**Sex**			
Male	21 (60.0%)	40 (60.6%)	1.000
Female	14 (40.0%)	26 (39.4%)	
**TNM stage**			
I, II	17 (48.6%)	29 (43.9%)	0.680
III, IV	18 (51.4%)	37 (56.1%)	

NOTE. Values are n (%) unless otherwise indicated.

Supplementary Table 8: Cox regression analysis for Relapse-Free Survival in CRC (colon) patients (Beijing cohort)

Colon cancer (n=101)	Univariate Analysis		Multivariate Analysis	
	Hazard ratio (95% CI)	P value	Hazard ratio (95% CI)	P value
DDX27 expression (high vs. low)	3.405 (1.177-9.849)	0.024	3.362 (1.157-9.771)	0.026
Age (>60 vs. <=60)	1.164 (0.547-2.477)	0.693	1.240 (0.572-2.688)	0.586
Male vs. female	1.052 (0.482-2.299)	0.898	1.085 (0.489-2.405)	0.842
Stage (III, IV vs.I, II)	1.854 (0.832-4.128)	0.131	1.730 (0.774-3.866)	0.182

Supplementary Table 9: Clinicopathologic Features of DDX27 Expression in Colorectal Cancer (colon) from TCGA.

TCGA (colon) Variable	Low DDX27 expression (n=39)	High DDX27 expression (n=320)	*P* value
**Age**, mean ± SD	71.3 ± 12.4	65.1 ± 12.9	0.004
**Sex**			
Male	22 (56.4%)	150 (46.9%)	0.309
Female	17 (43.6%)	170 (53.1%)	
**TNM stage**			
I, II	29 (74.4%)	170 (54.1%)	0.017
III, IV	10 (25.6%)	144 (45.9%)	

NOTE. Values are n (%) unless otherwise indicated.

Supplementary Table 10: Cox regression analysis for Relapse-Free Survival in CRC (colon) patients (TCGA cohort)

Colon cancer (n=359)	Univariate Analysis		Multivariate Analysis	
	Hazard ratio (95% CI)	P value	Hazard ratio (95% CI)	P value
DDX27 expression (high vs. low)	8.966 (1.243-64.699)	0.030	7.345 (1.014-53.203)	0.048
Age (>60 vs. <=60)	0.626 (0.375-1.043)	0.072	0.678 (0.388-1.185)	0.173
Male vs. female	1.493 (0.899-2.478)	0.122	1.595 (0.930-2.737)	0.090
Stage (III, IV vs.I, II)	2.519 (1.507-4.211)	<0.001	2.271 (1.344-3.838)	0.002

Supplementary Table 11: Cox regression analysis for Overall Survival in CRC patients (Shanghai cohort)

**Shanghai cohort (n=260)**	Univariate Analysis		Multivariate Analysis	
	Hazard ratio (95% CI)	P value	Hazard ratio (95% CI)	P value
DDX27 expression (high vs. low)	1.569 (1.091-2.255)	0.015	1.389 (0.962-2.005)	0.080
Age (>60 vs. <=60)	1.220 (0.802-1.857)	0.353	1.369(0.895-2.093)	0.147
Male vs. female	1.108 (0.769-1.597)	0.582	1.062 (0.735-1.534)	0.748
Stage (III, IV vs.I, II)	2.382 (1.653-3.431)	<0.001	2.382 (1.643-3.455)	<0.001

Supplementary Table 12: Cox regression analysis for Overall Survival in CRC patients (Shanghai cohort, stage I and II)

**Shanghai cohort stage I and II (N=156)**	Univariate Analysis		Multivariate Analysis	
	Hazard ratio (95% CI)	P value	Hazard ratio (95% CI)	P value
DDX27 expression (high vs. low)	1.789 (1.043-3.070)	0.035	1.860 (1.064-3.250)	0.029
Age (>60 vs. <=60)	0995 (0.532-1.863)	0.989	0.892 (0.471-1.692)	0.727
Male vs. female	0.841 (0.491-1.442)	0.530	0.756 (0.436-1.311)	0.320
Stage (II vs.I)	1.652 (0.658-4.152)	0.285	1.525 (0.600-3.879)	0.376

Supplementary Table 13. Primers used for quantitative PCR

ACTB-F	AGAGCTACGAGCTGCCTGAC
ACTB-R	AGCACTGTGTTGGCGTACAG
BIRC3-F	TTTCCGTGGCTCTTATTCAAACT
BIRC3-R	GCACAGTGGTAGGAACTTCTCAT
CCL20-F	TGCTGTACCAAGAGTTTGCTC
CCL20-R	CGCACACAGACAACTTTTTCTTT
CXCL3-F	CGCCCAAACCGAAGTCATAG
CXCL3-R	GCTCCCCTTGTTCAGTATCTTTT
hDDX27-F	AGGACATGAACCTTTCCCGC
hDDX27-R	CGGCAGTTTTACCTGTCCCA
NFKBIA-F	ACCTGGTGTCACTCCTGTTGA
NFKBIA-R	CTGCTGCTGTATCCGGGTG
TNF-F	AAGAGGGAGAGAAGCAACTACAGA
TNF-R	GGTGGAGCCGTGGGTCAG
TNFAIP3-F	TCAACTGGTGTCGAGAAGTCC
TNFAIP3-R	CAAGTCTGTGTCCTGAACGC
**For CHIP-PCR**	
BIRC3-CHIP-F	GTGTGTGTGGTTATTACCGC
BIRC3-CHIP-R	AGCAAGGACAAGCCCAGTCT
TNF-CHIP-F	CCACAGCAATGGGTAGGAGAATG
TNF-CHIP-R	TTCATGAAGCTCTCACTTCTCAG
